# Induced pluripotent stem cells: ex vivo models for human diseases due to mitochondrial DNA mutations

**DOI:** 10.1186/s12929-023-00967-7

**Published:** 2023-09-22

**Authors:** Chao Chen, Min-Xin Guan

**Affiliations:** 1 Center for Mitochondrial Biomedicine, The Fourth Affiliated Hospital, Zhejiang University School of Medicine, Hangzhou, Zhejiang China; 2 Division of Medical Genetics and Genomics, The Children’s Hospital, Zhejiang University School of Medicine and National Clinical Research Center for Child Health, Hangzhou, Zhejiang China; 3 Institute of Genetics, Zhejiang University International School of Medicine, 866 Yuhangtang Road, Hangzhou, 310058 Zhejiang China; 4 Zhejiang Provincial Key Laboratory of Genetic and Developmental Disorders, Hangzhou, Zhejiang China; 5 Key Lab of Reproductive Genetics, Ministry of Education of PRC, Zhejiang University, Hangzhou, Zhejiang China

**Keywords:** Mitochondria, Maternally inherited diseases, mtDNA mutations, iPSCs

## Abstract

Mitochondria are essential organelles for cellular metabolism and physiology in eukaryotic cells. Human mitochondria have their own genome (mtDNA), which is maternally inherited with 37 genes, encoding 13 polypeptides for oxidative phosphorylation, and 22 tRNAs and 2 rRNAs for translation. mtDNA mutations are associated with a wide spectrum of degenerative and neuromuscular diseases. However, the pathophysiology of mitochondrial diseases, especially for threshold effect and tissue specificity, is not well understood and there is no effective treatment for these disorders. Especially, the lack of appropriate cell and animal disease models has been significant obstacles for deep elucidating the pathophysiology of maternally transmitted diseases and developing the effective therapy approach. The use of human induced pluripotent stem cells (iPSCs) derived from patients to obtain terminally differentiated specific lineages such as inner ear hair cells is a revolutionary approach to deeply understand pathogenic mechanisms and develop the therapeutic interventions of mitochondrial disorders. Here, we review the recent advances in patients-derived iPSCs as ex vivo models for mitochondrial diseases. Those patients-derived iPSCs have been differentiated into specific targeting cells such as retinal ganglion cells and eventually organoid for the disease modeling. These disease models have advanced our understanding of the pathophysiology of maternally inherited diseases and stepped toward therapeutic interventions for these diseases.

## Background

Mitochondria are essential organelles for cellular metabolism and physiology in eukaryotic cells. The primary function of mitochondria acts as a powerhouse to generate the large quantities of ATP through oxidative phosphorylation (OXPHOS) [1]. Mitochondria also play the pivotal roles in regulating cellular calcium balance, adaptive thermogenesis, programmed cell death, and affecting inflammation and aging [2–4]. More than 1000 mitochondrial proteins encoded by nuclear genes are synthesized in the cytosol and transported to mitochondrial membranes or matrix [5], while 13 core components of OXPHOS complexes, encoded by mitochondrial DNA (mtDNA), are produced by mitochondrial translation machinery [6].

Human mtDNAs are referred as circular double-strand molecules with 16,569 base pairs, encoding 13 polypeptides for OXPHOS complexes, 2 ribosomal RNAs and 22 transfer RNAs required for the synthesis of polypeptides [7]. mtDNA replication is catalyzed by own replication machinery including DNA polymerase γ (Polγ), twinkle helicase and single stranded DNA binding protein (mtSSB) [8]. Unlike nuclear DNA, mtDNA is polyploid, which are multiple copies of mtDNA molecules within each cell. The numbers of mtDNA molecules vary greatly in different cell types and organs, from around 100 copies of mtDNA molecules in sperm to hundreds of thousands in oocytes [9–11]. During fertilization, mitochondria from sperm are selectively degraded in zygotes, thus mtDNA is inherited from mothers rather than fathers [12].

There is an inextricable connection between the polyploid mtDNA and genetic heterogeneity. Variations in mtDNA are present in either heteroplasmy (mixture of wild-type and mutated molecules) or homoplasmy (all mutated molecules) [13–18]. mtDNA mutations occur sporadically or arise due in large part to the high mutation rate of mtDNA and revealed the maternal inheritance [13–15]. The absence of abundant DNA repair system or protection of histones makes mtDNA more vulnerable to reactive oxygen species (ROS), generated in mitochondrial electron transport chain [1, 13]. mtDNA mutations also arise from defects in the biogenesis and mtDNA maintaince, reflecting the contribution of nuclear-encoded genes such as POLG to these processes, and in this case exhibit Mendelian inheritance [14, 17].

## Mitochondrial diseases

**Fig. 1:**
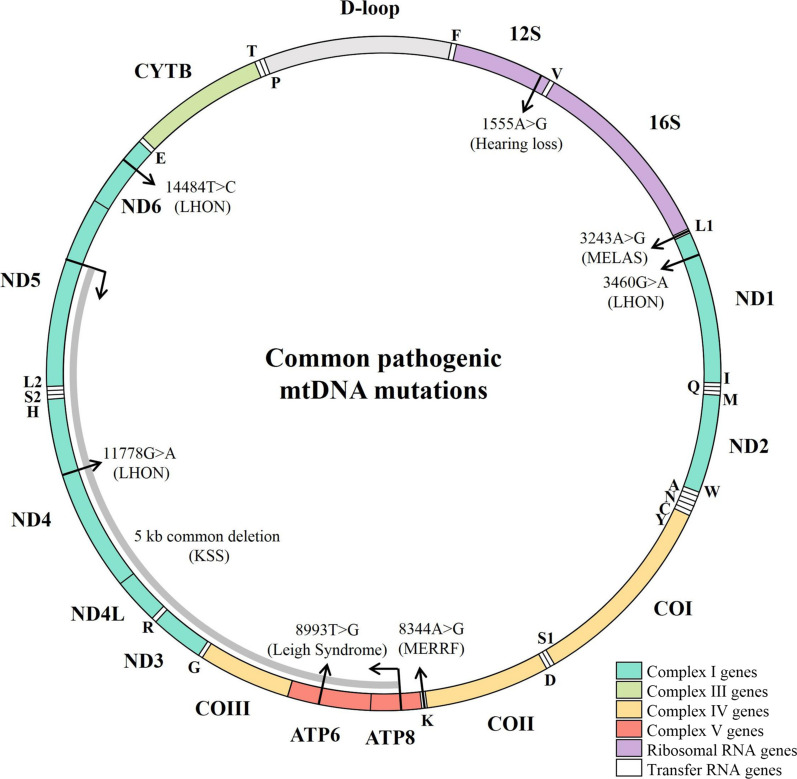
Mitochondrial genome and pathogenic mtDNA mutations. Human mitochondrial genome is shown as circular, double-stranded DNA molecule with annotations. The ribosomal RNA genes are shown in purple, while tRNA genes are shown in white and annotated with single letter abbreviations. The subunits of complex I (cyan), cytochrome b (green) of complex III, subunits of complex IV (yellow) and complex V (red) are denoted by the position along the mtDNA sequence, with the outer circle as the heavy chain, and inner circle as the light chain. The positions of pathogenic mtDNA mutations are marked by black arrows. *KSS* Kearns–Sayre syndrome, *LHON* Leber’s hereditary optic neuropathy, *MELAS* Mitochondrial Encephalomyopathy, Lactic Acidosis, and Stroke-like episodes, *MERRF* Myoclonic Epilepsy and Ragged Red Muscle Fibers

Mitochondrial diseases are a group of genetic disorders that affect any organ at any age, caused by mutations in nuclear and mitochondrial genes [1–3]. Mitochondrial diseases are often multi-systemic disorders, affecting tissues or organ with high energy demands, such as the nervous system, heart, and skeletal muscles [13–15]. Mitochondrial diseases also manifest single organ and tissue, such as ear and eye [18]. In this review, we focus on the mitochondrial diseases arising from mtDNA alterations (Fig. 1). These mtDNA alterations included mtDNA rearrangements such as deletions, inversions or duplications, point mutations, or copy number depletion. mtDNA large scale deletions are present in heteroplasmic form to cause clinical abnormalities including Kearns–Sayre syndrome (KSS), Pearson syndrome (PS), or Chronic progressive external ophthalmoplegia (CPEO) [13]. The heteroplasmic point mutations affected more than one organ or tissues including mitochondrial encephalomyopathy lactic acidosis, and stroke-like episodes (MELAS) associated tRNA^Leu(UUR)^ 3243A > G [19], myoclonic epilepsy associated with ragged-red fibers (MERRF) linked tRNA^Lys^ 8344A > G [20], Leigh syndrome-associated ATP6 8993T > G mutations [21]. By contrast, the homoplasmic or near homoplasmic mutations often manifest single organ or tissues, including Leber hereditary optic neuropathy (LHON)-associated ND4 11778G > A, ND1 3460G > A, and ND6 14484T > C [22], deafness-linked 12S rRNA 1555A > G and 1494C > T mutations [23, 24].

The heteroplasmic point mutations such as m.3243A > G mutation confer drastic biochemical defects and are by themselves enough to produce the clinical phenotype [1, 13,15, 19]. Individuals carrying the heteroplasmic mtDNA mutation often present with more than one initial clinical manifestation. The clinical phenotype of m.3243A > G mutation is highly complex and variable, ranging from asymptomatic to lethal phenotypes, partly depending on the level and distribution of m.3243A > G heteroplasmy across cells and tissues [25]. The development of phenotypes arising from these mutations depends on the proportion of mutated mtDNA molecules in the cells, surpassed a threshold level to maintain full OXPHOS function in the vulnerability of tissue or organ [1, 15]. The threshold effects vary among cells, tissues or organs, and the organs most affected with relatively low threshold levels are often those with high energy demand, such as the brain, muscles and heart [26]**.** Moreover, the elevated proportion of mutated mtDNA moecules across generation leads to genetic anticipation. Heteroplasmy dynamics might be positively or negatively modulated by mitophagy and other cellular contexts, while the underlying mechanisms have remained to be elucidated [27].

Homoplasmic mtDNA mutations often caused mild biochemical defects and manifested only one tissue or organs. Only some of matrilineal relatives bearing the mtDNA mutation(s) developed a clinical phenotype such as deafness, while other mutation carriers did not develop a disease phenotype. These mtDNA mutation(s) is by itself insufficient to produce the clinical phenotypes and other genetic and environmental factors such as nuclear modifier gene and aminoglycosides contributed to the development of clinical phenotype. In particular, there were marked variations in the penetrance and gender bias occurring in the LHON pedigrees carrying the mtDNA mutation, reflecting the complex etiology of this disease [28–30]. Mutations in the X-linked modifier *PRICKLE3* encoding a mitochondrial protein linked to the biogenesis of ATPase or autosomal recessive modifier *YARS2* encoding mitochondrial tyrosyl-tRNA synthetase interact with m.11778G > A mutation to cause optic neuropathy [28, 29]. The administration of aminoglycosides can induce or worsen hearing loss in these subjects carrying the m.1555A > G or m.1494C > T mutation [23, 24]. In the absence of aminoglycosides, a modifier allele (c.28G > T, p.Ala10Sser) in TRMU encoding methylaminomethyl-2-thiouridylate-methyltransferase related to tRNA modification interacts with m.1555A > G mutation to cause deafness [31, 32]. Moreover, mitochondrial genetic modifiers such as m.3394T > C, m.4435A > G, and m.3866 T > C mutations act in synergy with the m.11778G > A mutations to increase penetrance and expressivity of LHON [33–35].

Mitochondrial diseases present a tissue specificity characterized by the fact that even if a mtDNA mutation is present in all tissues, only some will be affected and induce a pathology. In fact, one mtDNA mutation is able to cause different clinical presentation, or same disease can result from different mtDNA mutations. Clinical presentations due to mtDNA mutations share neurological clinical features such as seizure, epilepsy, or myopathy, and non-neurological features such as cardiomyopathy, diabetes mellitus, or respiratory failure [18]. Tissue-specific effects of heteroplasmy mtDNA mutations reflect the differing bioenergetic requirements of different tissue [36]. The heterogeneity in the phenotypic expression of m.3243A > G mutation ranges from mild phenotype such as deafness to severe symptoms such as stroke-like episodes cardiomyopathy [19, 37]. The tissue specific defects of homoplasmic mtDNA mutations such as m.11778G > A or m.1555A > G mutation are due to the contribution of nuclear modifier genes or the use of aminoglycoside [28, 29, 31, 32, 38, 39]. However, the lack of appropriate cell and animal disease models has been significant obstacles for deep elucidating the pathophysiology of maternally transmitted diseases, especially for tissue-specific effects [19, 40].

## iPSCs

iPSCs can be induced to differentiate into nearly all cell types through guided stepwise differentiation protocols to recapitulate the embryonic development [41]. The induced pluripotency triggered great scientific enthusiasm and excitement for etiology research and cellular therapy applications [42]. In particular, the iPSCs can be generated from somatic cells by introducing four “Yamanaka factors”, Oct3/4, Sox2, c-Myc, and Klf4 [43, 44]. These iPSCs derived from human patient cells have provided the excellent disease models to investigate the etiology in affecting organs or tissues, subtly bypassing the impracticality to conduct biopsy on eyes or inner ears. Since the original discovery, the follow-up studies reported plentiful of reprogramming strategies including viral or non-viral system delivering distinct combinations of exogenous transcription factors, RNAs, and even small-molecule compounds [45].

Towards appropriate patient derived iPSCs, the source of somatic cells, transcription factors, reprogramming enhancers, and the delivery strategies should all be taken into consideration. Fibroblasts are most used source of cells for reprogramming for the well-established skin biopsy and culturing techniques [44, 46]. Peripheral blood mononuclear cells (PBMCs) and cells derived from urine, easily available in medical examinations, were also reported to be reprogrammed to iPSCs [47, 48], as well as CD34^+^ hematopoietic stem cells [49], T-lymphocytes [50], and keratinocytes [51]. Once the cell type was chosen, the set of reprogramming factors should be compatible. Variations of the combinations of reprogramming factors to optimize the efficiency for generating iPSCs have been essential issues to better understand induced pluripotency [52]. Epigenetics changes occurred during the reprogramming process. Hence small-molecule chemicals affecting epigenetics act as enhancers for reprogramming, for example, vitamin C inducing the demethylation of DNA [53], valproic acid and sodium butyrate inhibiting histone deacetylase [54, 55], and 5-azacytidine (AZA) inhibiting DNA methyltransferase [56]. Moreover, the choice of delivery strategy is the selection of vehicle to introduce the reprogramming factors to designated cells, affecting the efficiency and safety. Integrative strategies including retrovirus, lentivirus, and transposon were used in the early studies, yet raising the concerns of carcinogenicity [43, 44, 46, 57, 58]. Most recently, nonintegrative strategies including adenovirus, sendai virus and episomal vectors have been developed [59–61]. Furthermore, direct delivery of proteins, mRNAs, self-replicative RNAs, miRNAs, or small-molecule compounds have been used sufficiently for reprogramming [62–66].

The use of human-iPSC (hiPSC) derived from patients to obtain terminally differentiated specific targeting cells such as neurons and cardiomyocytes is a revolutionary approach to understanding pathogenic mechanisms and to develop effective therapeutic strategies for human diseases [67]. In particular, the iPSCs technology has offered great opportunity to shed new light on the threshold effect, incomplete penetrance, and tissue specificity of mitochondrial diseases. It should be taken into consideration in modelling mitochondrial diseases whether the status of mtDNA mutations is altered after reprogramming from patient cells to iPSCs, or after the differentiation to target cells [68]. The long-term monitor of mtDNA should be included to avoid de novo mtDNA mutations when culturing or manipulating the iPSCs.

## iPSC models for mitochondrial diseases

**Fig. 2:**
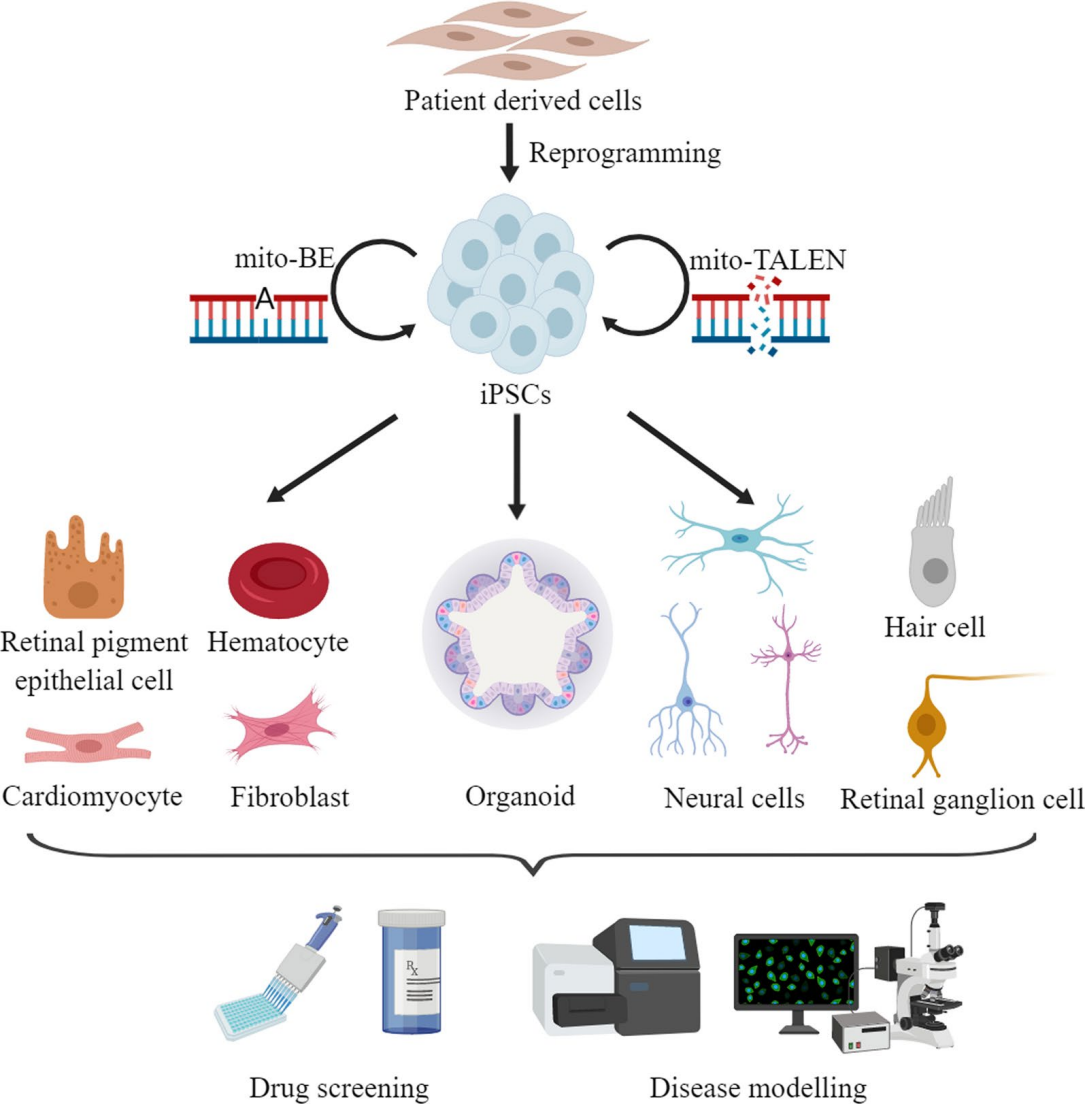
iPSC models for human diseases due to mtDNA alterations. In modelling mtDNA diseases, patient derived cells are firstly reprogrammed to iPSCs. With mitochondrial base editing and mito-TALEN, the manipulating of mtDNA in iPSCs is achievable. The patient derived iPSCs and genetically corrected iPSCs are differentiated to distinct types of targeting cells to investigate the pathophysiology and to develop the therapeutic intervention approaches for these diseases

A number of patient-derived iPSCs models for mitochondrial diseases have been established (Fig. 2). In this review, we summarized these patient-specific iPSCs models for mitochondrial disorders due to mtDNA large deletions, point mutations in protein-coding genes, tRNA and 12S rRNA genes (Table 1). These patients-derived iPSCs have been differentiated into specific targeting cells such as retinal ganglion cells (two-dimensional differentiation) and eventually organoid (three-dimensional differentiation) for disease modeling. These models have advanced our deep understanding the pathogenic mechanism, especially in tissue specific effects, and stepped toward therapeutic interventions for these diseases.

**Table 1:** iPSC models of mtDNA mutation induced diseases

Syndrome/disease	Locus in mtDNA or nDNA	Mutation	Type of mtDNA mutation	Effects on			References
				Efficiency of reprogramming	Maintenance of pluripotency	Differentiation capacities	
Pearson syndrome		Large scale deletion	Het	Low efficiency	Not affected	Differentiated to hematopoietic cells with defective functions	[75, 76]
Kearns Sayre syndrome		Large scale deletion	Het	N/A	N/A	N/A	[77, 78]
MELAS	tRNA^Leu(UUR)^	m.3243A > G	Het	Low efficiency	Not affected	Differentiated to cardiomyocytes, neuronal cells, and RPEs with defective functions	[79, 81, 82, 84–91, 93]
	ND5	m.13513G > A	Het	Not affected	Not affected	Incapable of αSMA-positive cells differentiation, and abnormal TUBB3-positive cells differentiation	[80, 92]
	tRNA^Trp^	m.5541C > T	Het	N/A	N/A	Differentiated to neuronal cell and skeletal muscle with impaired neuronal maturation	[83]
MERRF	tRNA^Lys^	m.8344A > G	Het	Low efficiency	Not affected	Differentiated to cardiomyocytes, NPCs, and inner ear HC-like cells with defective functions	[101, 102]
Leigh syndrome	ATP6	m.8993 T > G	Het	N/A	Not affected	Differentiated to skeletal muscle cells and cardiomyocytes with defective functions/Impaired differentiation potential in high mutation rate cells	[82, 108, 109, 111]
		m.9185 T > C	Hom	N/A	Not affected	Differentiated to NPCs with defective functions	[107, 109]
		m.9154C > T	Het	Not affected	Not affected	Differentiated to motor neurons with defective functions	[112]
	ND5	m.13513G > A	Het	N/A	Not affected	Inefficient differentiation to cardiomyocytes; differentiated to neurons with defective functions	[82, 110]
LHON	ND1/ND6	m.4160T > C/m.14484T > C	Hom	N/A	Not affected	Differentiated to RGCs with defective functions	[113]
	ND4	m.11778G > A	Hom	N/A	Not affected	Differentiated to RGCs and neurons with defective functions	[114–117]
	ND1	m.3460G > A	Hom	N/A	Not affected	Differentiated to neurons with defective functions	[117]
	ND4/PRICKLE3	m.11778G > A/c.157C > T	Hom	N/A	Not affected	Differentiated to RGCs with defective functions	[125]
	ND4/YARS2	m.11778G > A/c.572G > T	Hom	N/A	Not affected	Differentiated to RGCs with defective functions	[126]
Hearing loss	12S rRNA/TRMU	m.1555A > G/c.28G > T	Hom	N/A	Not affected	Differentiated to inner ear HC-like cells with defective functions	[134]

*Het* heteroplasmy, *Hom* homoplasmy, *N/A* not available, *MELAS* mitochondrial encephalomyopathy lactic acidosis, and stroke-like episodes, *MERRF* myoclonic epilepsy associated with ragged-red fibers, *LHON* Leber hereditary optic neuropathy, *ND1/4/5/6* mitochondrially encoded nadh:ubiquinone oxidoreductase core subunit 1/4/5/6, *PRICKLE3* prickle planar cell polarity protein 3, *YARS2* mitochondrial tyrosyl-tRNA synthetase, *TRMU* mitochondrial tRNA 2-thiouridylate, *RPE* retinal pigment epithelial, *αSMA* alpha smooth muscle actin, *TUBB3* tubulin beta 3 class III, *NPC* neural progenitor cell, *HC* hair cell, *RGC* retinal ganglion cell

### Kearns–Sayre syndrome (KSS) and Pearson syndrome (PS)

Several patient-derived iPSCs for KSS and PS with mtDNA deletions have been established and characterized. KSS and PS are the multisystem mitochondrial diseases caused by large-scale mitochondrial DNA deletions, especially the common 4,977-bp deletion [69–74]. KSS is a rare neuromuscular disorder, characterized by progressive external ophthalmoplegia, pigmentary retinopathy, with additional cardiac conduction defects, or ataxia [69, 70, 74], while PS is a multisystem mitochondrial disease with bone marrow failure [71–73]. iPSC derived from PS and KSS patients carrying the heteroplasmy mtDNA deletions have been established. The heteroplasmy level of mutant mtDNA in these iPSC lines ranged from high levels of mutation to normal mitochondrial DNA during cell expansion of iPSCs generated from patients. PS-iPSCs carrying a high burden of deleted mtDNA exhibited defects in growth, mitochondrial ultrastructure, oxidative phosphorylation and hematopoietic phenotype when differentiated in vitro, compared to isogenic iPS cells without deleted mtDNA [75, 76]. Furthermore, hiPSC derived from KSS patients with 7.3 kb mtDNA deletion were generated [77, 78]. Interestingly, some iPSCs from these KSS or PS patients showed normal differentiation toward to target tissue or organ without any mtDNA deletions over passages [75–78]. These open the new avenues to create isogenic mutation free iPSC with absent or very low level of expression of mtDNA deletion for future cell replacement therapies.

### MELAS

The hiPSCs models for MELAS syndrome, caused by the heteroplasmic mtDNA mutation(s), covered disease modelling and therapeutic applications [79–94]. MELAS syndrome is one of the most frequent maternally inherited mitochondrial disorders, with broad manifestations including stroke-like episodes, dementia, epilepsy, lactic acidemia, myopathy, recurrent headaches, hearing loss, diabetes, and short stature [19, 94, 95]. The tRNA^Leu(UUR)^ 3243A > G mutation is the most prevalent MELAS-associated mtDNA mutation [94, 95], while other MELAS-associated mtDNA mutations included the tRNA^Leu(UUR)^ 3271T > C, tRNA^Trp^ 5541C > T and ND5 13513G > A mutations [94, 95]. In particular, the m.3243A > G mutation caused pleiotropic defects in tRNA^Leu(UUR)^ metabolism, ultimately impairing OXPHOS and mitochondrial functions [96, 97]. During the programming, the iPSC lines underwent an mtDNA bottleneck with bimodal segregation and carried the mutation at variable heteroplasmy, ranging from nearly homoplasmy to mutation-low or mutation-free [79–82]. Those iPSCs-derived specific types of cells such as neuron, cardiomyocytes and retinal pigment epithelium (RFE) cells have made possible the study of not only common pathophysiological alterations but also cell-specific defects in the disease [79–83]. MELAS iPSC-derived neurons harboring high m.3243A > G mutation load showed defective OXPHOS function, increasing ROS production, aberrant mitophagy and impaired neuronal network functionality and synchronicity [79, 81]. MELAS iPSC bearing nearly homoplasmic m.5541C > T mutation exhibited defective neuronal differentiation but without apparent impairment of neural progenitors or skeletal muscle cells [82]. Furthermore, MELAS iPSC-derived neurons with high m.3243A > G levels displayed abnormal electrophysiological properties: reduced mean firing rate, reduced network burst rates and increased random spike events outside of network bursts, while neurons with intermediate heteroplasmy levels (~ 30%) were relatively normal, indicative of the threshold effect for mtDNA mutations [81]. Other MELAS iPSC-derived cell types included the endothelial cells displaying pro-atherogenic properties and RPE cells exhibiting dysfunctional phagocytosis [84–86]. In particular, these RPE cells revealed abnormal mitochondria and melanosomes, marked functional defects in phagocytosis of photoreceptor outer segments, and dysregulated autophagic activity [84, 85, 89]. Moreover, motor neurons bearing the m.3243A > G mutation exhibited defective mitochondrial respiration and developmental deficit, evidenced by elevating Notch signaling that maintained the stem cell identity in NPCs and inhibited neurite outgrowth in neurons [90, 98, 99].

The hiPSC model for MELAS can be used for developing therapeutic interventions. The levels of heteroplasmic m.3243A > G and m.13513G > A mutations in iPSCs were efficiently reduced by mito-TALEN approach to reduce mutant mtDNA loads and restore oxidative phosphorylation function in mitochondrial diseases [91, 92]. Moreover, a small-molecule compound tryptolinamide (TLAM) activated mitochondrial respiration in fibroblasts from patient-derived iPSCs carrying the m.3243A > G mutation and rescued the defect in neuronal differentiation of iPSCs with high ratio of mutation [93].

### MERRF

MERRF (myoclonic epilepsy with ragged red fibers) is a rare syndromic mitochondrial disorder, mainly caused by the heteroplasmic tRNA^Lys^ 8344A > G mutation [20, 100]. The primary clinical features in this disorder are myoclonus, dementia, muscular weakness, ataxia and ragged-red fibers in the muscle, and other secondary clinical manifestations included peripheral neuropathy, renal dysfunction or cardiomyopathy or hearing impairment [100]. These iPSCs derived from MERRF patients carrying the m8344A > G mutation were differentiated into neuron like cells, which allow the study of these diseases in one of the most affected cellular types [101–103]. In particular, neural progenitor cells (NPCs) derived from iPSCs from MERRF patients bearing the m.8344A > G mutation exhibited the pathophysiological features previously observed in other disease models, such as an impaired mitochondrial structure and function, increased the level of ROS production, altered antioxidant enzyme expression (101). Furthermore, induced neurons derived from iPSc reprogrammed from skin fibroblasts of MERRF patients harboring the m.8344A > G mutation showed pathophysiological features including impaired mitochondrial morphology and function, reduced membrane potentials, elevated ROS levels, autophagy flux disruption and increased mitophagy [103]. Moreover, inner ear hair cells derived from iPSCs carrying the m.8344A > G mutation displayed significantly elevated ROS levels, more single cilia with a shorter length or fewer stereociliary bundle-like protrusions, and altered transcriptome [102].

### Leigh syndrome

Leigh Syndrome (LS) is a rare, inherited neurometabolic disorder that mainly affects the central nervous system, caused by mutations in mitochondrial DNA or nuclear genes encoding mitochondrial proteins [104, 105]. The heterogeneous neurological symptoms include hypotonia, developmental delay, ataxia, seizures, dystonia, polyneuropathy, nystagmus, and optic atrophy [104]. The LS-causative mtDNA mutations included hetroplasmic ATP6 8993 T > G and 9185T > C and ND5 13513G > A mutation [104–106]. LS iPSC-derived patients carrying the m.8993T > G, m.9185T > C or m.13513G > A mutation were differentiated into neural progenitors, neurons, and brain organoids [107–112]. Neural cells differentiated from these LS iPSCs bearing the m.8993T > A or 9185T > C mutation revealed deficiencies in bioenergetics, mitochondrial morphologies, calcium homeostasis and increased susceptibility to glutamate toxicity [81, 107–109]. Neurons derived from patients carrying the heteroplasmic m.13513G > A mutation exhibited defects in OXPHOS function and calcium signaling [110]. Furthermore, brain organoids carrying the m.8993T > G mutation displayed developed abnormally, showing reduced size and altered cortical architecture [111]. Motor neurons from iPSCs bearing the m.9154C > T mutation were compatible with motor neuron identity and function to determine the threshold effect of heteroplasmic ATP6 mutation in reprogramming, Notch hyperactivation and motor neuron metabolism [112].

### LHON

The LHON disease model generated by iPSCs derived from patients to obtain terminally differentiated RGCs allowed us to elucidate the tissue-specific effects, and develop effective therapeutic strategies for RGC disorders [113–117]. LHON is the most common maternally inherited eye disease characterized by selective loss of RGCs and their axons, which leads to rapidly progressive bilateral vision loss in young adults [118, 119]. The homoplasmic or nearly homoplasmic ND4 11778G > A, ND1 3460G > A, and ND6 14484T > C mutations, which affects the essential component of complex I (NADH: ubiquinone oxidoreductase), accounted for the majority of LHON cases worldwide [118–123]. The primary defects in the LHON-linked mtDNA mutation were the complex I deficiencies, thereby causing the respiratory deficiency, diminishing ATP synthesis, an increasing generation of reactive oxygen species and impaired apoptosis and mitophagy [29, 33, 35, 121, 122]. The incomplete penetrance and gender bias in patients presenting with optic neuropathy suggested nuclear modifier genes such as X-linked modifier necessary for the phenotypic manifestation of LHON-associated mtDNA mutations [28, 29, 116, 118, 119, 124]. Several LHON disease models were generated by iPSCs derived from patient’s cells bearing the m.11778G > A, m.14484T > C or m.3460G > A mutation [113–117, 125, 126]. These patients-derived iPSCs from LHON families included asymptomatic and symptomatic subjects carrying the mtDNA mutation(s) and control subjects. Those iPSCs were differentiated into neural progenitor cells and subsequently induced RGC-like cells using a stepwise differentiation procedure. These RGC-like cells derived from revealed more drastic reductions in oxygen consumption rates, levels of mitochondrial ATP and increasing productions of reactive oxygen species than those in other cell models such as cybrids [115, 117, 121, 122, 125, 126]. These mitochondrial dysfunctions increased susceptibility to apoptotic process for RGC degenerations [115, 125, 126]. These RGC-like cells derived from symptomatic individual harboring both mtDNA and nuclear modifier *PRICKLE3* or *YARS2* mutations exhibited greater defects in neuronal differentiation, morphology including reduced area of soma, numbers of neurites and shortened length of axons, electrophysiological properties than those in RGC-like cells derived from asymptomatic subjects bearing mtDNA mutation(s) [125, 126]. The nuclear modifier allele such as YARS2 c.572G > T mutation in iPSC lines from a syndromic individual can be corrected by CRISPR/Cas9. Strikingly, the genetic correction of YARS2 c.572G > T mutation led to morphologic and functional recovery of patient-derived RGC-like cells [126]. These findings provide new insights into pathophysiology of LHON arising from RGC-specific mitochondrial dysfunctions and a step toward therapeutic interventions for this disease.

### Maternally inherited deafness

mtDNA mutations are associated with the maternal transmission of syndromic deafness (hearing loss with other medical problems such as diabetes) and nonsyndromic deafness (hearing loss is the only obvious medical problem). The 12S rRNA 1555A > G and 1494C > T mutations have been associated with both aminoglycoside-induced and nonsyndromic deafness in many families worldwide [23, 24, 39]. The syndromic deafness-associated mutations included the MELAS-associated tRNA^Leu(UUR)^ 3243A > G mutation and MERRF-associated tRNA^Lys^ 8344A > G mutation and maternally inherited diabetes and deafness (MIDD)-associated tRNA^Glu^ 14692A > G mutation [19, 20, 127]. The nonsyndromic deafness-associated mtDNA mutations included tRNA^Ser(UCN)^ 7511T > C, tRNA^His^ 12201T > C, tRNA^Asp^ 7551A > G, tRNA^Ile^ 4295A > G andtRNA^Cys^ 5783C > T mutations [128–132]. In particular, the m.1555A > G mutation is a primary factor underlying the development of hearing loss and TRMU allele (c.28G > T, p.Ala10Sser) encoding tRNA thiouridylase interact with m.1555A > G mutation to cause hearing loss [31, 32]. Hsu et al. generated an iPSC line from the peripheral blood mononuclear cells derived from a hearing-impaired patient carrying the m.1555A > G mutation [133]. Chen et al. generated iPSCs from lymphoblastoid cell lines derived from members of an Arab–Israeli family (asymptomatic individual carrying only m.1555A > G mutation, symptomatic individual bearing both m.1555A > G and TRMU c.28G > T mutations, and control subject) [134]. The c.28G > T mutation in iPSC lines from a hearing-impaired subject harboring both m.1555A > G and TRMU c.28G > T mutations was corrected by CRISPR/Cas9 [134]. Four iPSC lines were differentiated into otic epithelial progenitors (OEPs) and subsequent inner ear hair cell-like cells (HCs). The iPSCs bearing the m.1555A > G mutation displayed mild defects in differentiation and resultant HC-like cells displayed mild defects in morphology and electrophysiological properties. Those HC-like cells harboring m.1555A > G and TRMU c.28G > T mutations exihibited greater defects in the development, morphology and functions than those in cells bearing only m.1555A > G mutation. Genetic correction of TRMU c.28G > T mutation resulted in morphologic and functional recovery of patient derived HC-like cells [134]. These findings provided new insights into pathophysiology of deafness arising from HC-specific mitochondrial deficiencies and a step toward therapeutic interventions for this disease.

## Diversity of mtDNA variants (non-pathogenic) in the iPSCs models

The genetic and functional integrity of iPSCs is an important consideration towards high-quality therapeutic application. Aside from the pathogenic mtDNA mutations, mtDNA variations accumulated later in life affect mitochondrial functions. Perales-Clemente et al. showed that one possible cause of intra-person iPSC variability was the impaired mitochondrial respiration detected in iPSC-derived cardiomyocytes, due to expanded mtDNA mutations non-related with human diseases [135]. Kang et al. examined the accumulation of mtDNA mutations in iPSCs derived from young and elderly individuals [136].These mtDNA mutations randomly arose within cultured cells and resulted in respiratory deficiency. The frequency of mutations in iPSCs increased with age [136]. In support of these observations, by deep-sequencing the iPSCs, increasing numbers of variants in mtDNA were detected during time in culture [68, 137, 138]. Wei et al. reported a rather high mutation rate at 8.62 × 10^–5^/base pair of iPSC mtDNAs [68]. These variants indicate appearance of chromosomal abnormalities [137]. The mutation rates of mtDNA are inherently higher than that of nuclear DNA. One of the possible hypotheses is that mtDNA is extensively exposed to high levels of ROS, and the fidelity of mitochondrial DNA polymerase gamma is inadequate in mtDNA replication. Specific mtDNA variants lead to faster replication causing heteroplasmy shifts over time. During cytokinesis, asymmetric partitioning of mtDNA variants is also hypothesized to affect the segregation of variants [26]. Furthermore, de novo mutations occurring in the mtDNA of cultured iPSCs were reported to encode neoantigens that provoke specific immune responses, suggesting that iPSCs and their derivatives are not inherently immunologically available for autologous transplantation [138]. Recently, the Schrepfer group showed that hypoimmunogenic iPSCs with inactivated major histocompatibility complex (MHC) class I and II genes, and over-expression of CD47 lost their immunogenicity [139]. Derivatives of these hypoimmunogenic iPSCs evaded immune rejection in vivo, providing a more successful and more economically viable strategy for regenerative medicine [139]. These results highlight the necessity of monitoring mtDNA mutations in iPSCs and immunologically properties to ensure the effectiveness in disease studies and clinical applications.

## iPSCs for therapeutic interventions for mitochondrial diseases

**Fig. 3:**
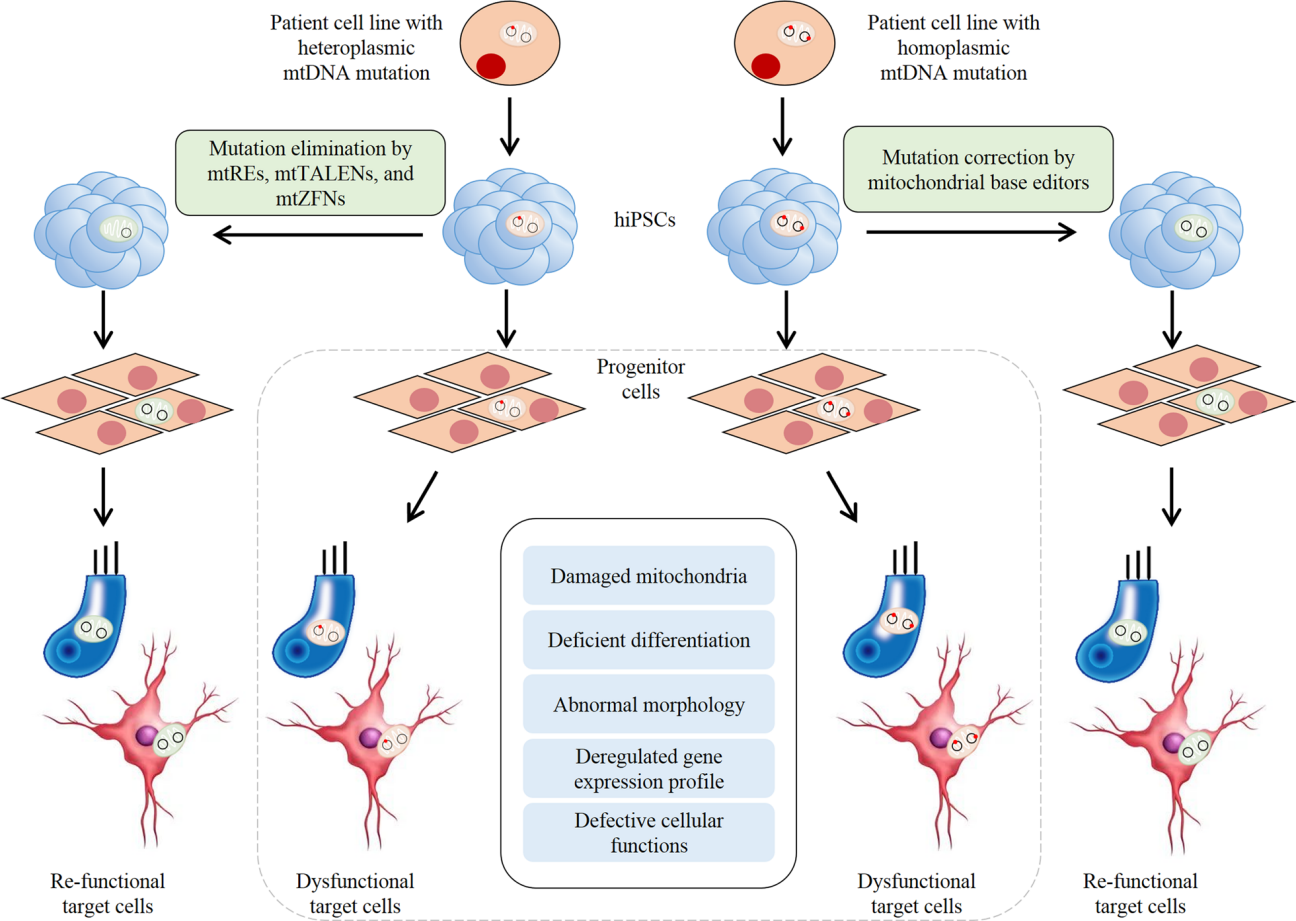
Therapeutic approaches combining iPSCs with mtDNA gene editing technology. For iPSCs derived from patients carrying heteroplasmic mtDNA mutation, mitochondrial restriction endonucleases (mtREs), mitochondrial-targeted transcription activator-like effector nucleases (mtTALENs), and zinc-finger nucleases (mtZFN) are utilized to eliminate the mutant mtDNA molecules. For iPSCs derived from patients bearing homoplasmic mtDNA mutation, mtDNA base editing technology is preferred to correct the mutations. After gene editing, the corrected iPSCs differentiate to target cells with remodeling cellular functions. The black circles indicate mtDNA. The red dots on black circles denote mtDNA mutations. The green color of mitochondria indicates normal condition, and the pink color of mitochondria show damaged function. Other colors of cells do not indicate any information

These iPSC patient-derived disease models can be used for developing therapeutic intervention strategy for mitochondrial diseases. Mitochondria targeting restriction endonucleases (mitoREs), TALENs (mitoTALENs), and ZFNs (mtZFNs) have been used to eliminate mutated mtDNA molecules in the iPSCs or organoid bearing heteroplasmic mtDNA mutations including the m.3243A > G mutation (Fig. 3) [91, 92, 140]. The base editing for mtDNA in iPSCs may enable to generate mitochondrial disease model and develop potential therapeutic intervention. In fact, the conventional CRISPR/Cas systems relying on small guide RNAs (sgRNAs) are incompatible with mitochondria, because sgRNAs are hardly imported into mitochondria [140]. The Liu group engineered inactive split-DddA halves until brought together on target DNA in mitochondria by adjacently bound programmable DNA-binding proteins (TALE) [141]. The employment of this RNA-free DddA-derived cytosine base editors (DdCBEs) achieved in catalysing C•G-to-T•A conversions in human mtDNA. Moreover, the Kim group engineered deoxyadenosine deaminase derived from the bacterial TadA protein with TALE to catalyze A-to-G conversions [142], extending the application range for mtDNA base editing. The combination of iPSC technology with these gene editing technology will provides the powerful tools to generate disease models and develop the effectively therapeutic approaches for these diseases.

## Conclusions

Despite the discovery of maternally inherited diseases caused by mtDNA mutations 35 years ago, there are many major challenges in elucidating the pathophysiology and developing the treatments for these disorders. To overcome these difficulties, many groups have turned to hiPSCs derived from patients carrying the mtDNA mutation(s) to model mitochondrial diseases. Here, we review the recent advances in patients-derived iPSCs as mitochondrial disease models. Those patients-derived iPSCs have been differentiated into specific cells (2-dimension differentiation) such as retinal ganglion cells, neuronal cells, muscle, cardiac cells and organoids (3-dimension differentiation). The iPSC-based modeling, in combining with mtDNA base editing technology, lead to deep elucidation of pathologic mechanism and opening new revenue to interventional strategies for these disorders.

## Data Availability

Not applicable.

## Abbreviations

mtDNA: Mitochondrial DNA
iPSCs: Induced pluripotent stem cells
OXPHOS: Oxidative phosphorylation
ROS: Reactive oxygen species
POLG: DNA polymerase gamma
KSS: Kearns–Sayre syndrome
PS: Pearson syndrome
CPEO: Chronic progressive external ophthalmoplegia
MELAS: Mitochondrial encephalomyopathy lactic acidosis, and stroke-like episodes
MERRF: Myoclonic epilepsy associated with ragged-red fibers
LHON: Leber hereditary optic neuropathy
ATP6: Mitochondrially encoded atp synthase membrane subunit 6
ND4: Mitochondrially encoded nadh:ubiquinone oxidoreductase core subunit 4
ND1: Mitochondrially encoded nadh:ubiquinone oxidoreductase core subunit 1
ND6: Mitochondrially encoded nadh:ubiquinone oxidoreductase core subunit 6
PRICKLE3: Prickle planar cell polarity protein 3
YARS2: Mitochondrial tyrosyl-tRNA synthetase
TRMU: Methylaminomethyl-2-thiouridylate-methyltransferase
Oct3/4: Octamer-binding transcription factor 3/4
Sox2: SRY-box transcription factor 2
c-Myc: MYC proto-oncogene, BHLH transcription factor
Klf4: Kruppel-like factor 4
PBMCs: Peripheral blood mononuclear cells
AZA: 5-Azacytidine
NPCs: Neural progenitor cells
ND5: Mitochondrially encoded nadh:ubiquinone oxidoreductase core subunit 5
CO3: Mitochondrially encoded cytochrome c oxidase III
CYB: Mitochondrially encoded cytochrome b
RPE: Retinal pigment epithelial
DAPT: (N-[N-(3, 5-difluorophenacetyl)-l-alanyl]-s-phenylglycinet-butyl ester)
TALEN: Transcription activator-like effector nuclease
TALM: Tryptolinamide
SCNT: Somatic cell nuclear transfer
RGC: Retinal ganglion cells
KIF5A: Kinesin family member 5A
MIDD: Maternally inherited diabetes and deafness
OEP: Otic epithelial progenitor
HC: Hair cell
MHC: Major histocompatibility complex
DdCBE: DddA-derived cytosine base editor
